# Impact of neonatal cardiac receive-array coil on SNR and CNR in newborn infants

**DOI:** 10.1186/1532-429X-13-S1-P215

**Published:** 2011-02-02

**Authors:** Anna E Finnemore, Anthony N Price, Giuliana Durighel, Jo V Hajnal, Alan M Groves

**Affiliations:** 1 Imperial College, London, UK

## Objective

To compare signal to noise ratio (SNR) and contrast to noise ratio (CNR) for myocardium and blood during cardiac MR imaging in the newborn using 2 different receive-array coils. Paediatric Body/Cardiac (Paed-Body) and Flex-M coils were compared under a clinical investigation agreement with Philips.

## Background

CMR assessments of cardiac function at 3.0 Tesla have been shown to provide more complete data with greater reproducibility than existing echocardiographic methods in newborns [1]. However signal to noise is at a premium in preterm infants. Further advances in image quality and spatial resolution are dependent on maximizing available signal. Prior neonatal CMR imaging has been performed using a 2-channel Flex-M receiver coil. However an 8-channel coil for neonatal imaging at 3T is now available.

## Methods

**Figure 1:**
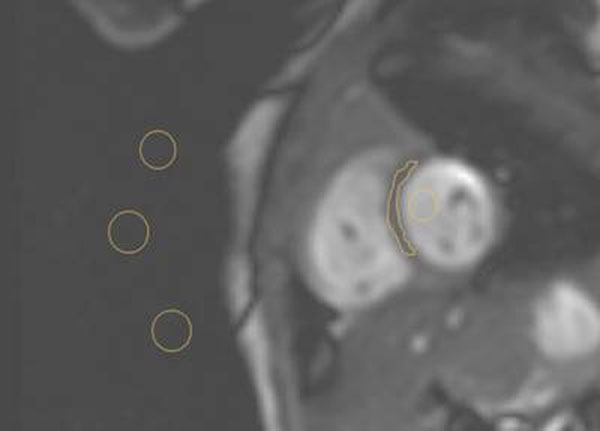
Location of measures of signal and background noise from a short axis image in a newborn infant

Cardiac MRI scans from 38 newborns of corrected gestation 26-41 weeks were retrospectively analysed in 2 weight-matched groups. All scans were performed in a Philips 3T MRI Scanner. Cine images were acquired with resolution 1.5/1.5/5mm. In 19 infants a 2-channel Flex-M coil was placed above and below the chest; in 19 an 8-channel Paed-Body receive-array coil was placed around the infant. Myocardial and blood signal intensity (SI) were measured at the intraventricular septum and left ventricular cavity respectively, at end-diastole from the mid-ventricular level of a short axis stack(Figure 1). Noise was taken as the mean of three measures of standard deviation in air surrounding the infant. SENSE and CLEAR techniques were not used. Images were analysed offline(CMR tools, CISL, London). SNR was taken as SI/noise. CNR was taken as (SI_blood_-SI_myocardium_)/noise.

## Results

**Figure 2:**
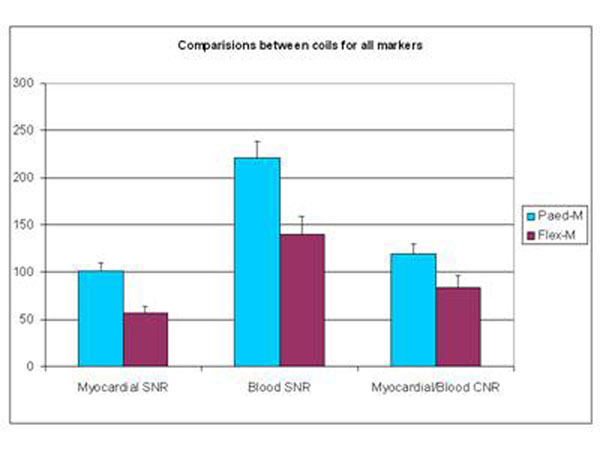
Comparison of myocardial and blood SNR and CNR using Paed-Body and Flex-M coils in 38 newborn infants

The 2 groups of infants were matched for weight (1923vs1911 grams, p=0.96). The Paed-Body coil produced significantly higher SNR in myocardium (101.4vs56.6, p=0.0003), SNR in blood (221.2vs139.9 p=0.003) and CNR (119.8vs83.5 p=0.03) (Figure 2). These findings persisted when only infants with weight <2000grams were assessed (myocardial SNR 114.2vs53.7 p=0.002, blood SNR 240.8vs131.1 p=0.01, CNR 126.6vs77.4 p=0.05).

## Conclusions

The Paed-Body receive-array coil produces significant increases in SNR and CNR when performing cardiac MR imaging in newborn infants including those weighing less than 2000g.
